# Immediate anxiety-reducing effects of multisensory virtual reality reminiscence therapy in older adults with a wide range of cognitive functions: a pilot study using electrophysiological measures

**DOI:** 10.3389/fnagi.2026.1958403

**Published:** 2026-10-08

**Authors:** Keita Ueno, Kazuyuki Niki, Hiroyuki Tanaka, Takeshi Okuda, Daisuke Iwai, Kosuke Sato, Yasuo Naito, Toshinori Ito, Ryouhei Ishii

**Affiliations:** 1 Department of Occupational Therapy, Morinomiya University of Medical Sciences, Osaka, Japan; 2 Graduate School of Rehabilitation Science, Osaka Metropolitan University, Osaka, Japan; 3 Department of Clinical Pharmacy Research and Education, The University of Osaka Graduate School of Pharmaceutical Sciences, Osaka, Japan; 4 Social Welfare Corporation Misasagikai, Osaka, Japan; 5 Graduate School of Engineering Science, The University of Osaka, Osaka, Japan; 6 Department of Science and Engineering, Otemon Gakuin University, Osaka, Japan; 7 Osaka Center for Cancer and Cardiovascular Disease Prevention, Osaka, Japan; 8 Department of Psychiatry, The University of Osaka Graduate School of Medicine, Osaka, Japan

**Keywords:** anxiety, dementia, electroencephalography, frontal alpha asymmetry, mild cognitive impairment, multisensory stimulation, reminiscence therapy, virtual reality

## Abstract

**Introduction:**

Anxiety is a prevalent behavioral and psychological symptom of dementia (BPSD) in older adults with cognitive impairment, severely diminishing their quality of life. Although reminiscence therapy (RT) is a promising non-pharmacological intervention, objective neurophysiological methods to evaluate its immediate emotional impact remain unexplored. This pilot study proposed “Smart Reminiscence Therapy (SRT)” – a novel virtual reality (VR) system integrated with synchronized multisensory stimuli – and evaluated its immediate anxiety-reducing effects and the utility of electrophysiological measures.

**Methods:**

A single-session, single-arm pre-post exploratory design was adopted. Twenty older adults (aged 65 years or older) at a day service center, with Mini-Mental State Examination-Japanese (MMSE-J) scores of 11–30, participated. The intervention consisted of a 10-min SRT session featuring a 1960s Japanese townscape. The primary outcome was objective resting-state eye-closed frontal alpha asymmetry (FAA) via a wearable EEG device, while subjective state anxiety via the State-Trait Anxiety Inventory (STAI) was evaluated as a secondary outcome.

**Results:**

After excluding three participants with excessive artifacts, 17 participants (mean age: 85.9 ± 5.9 years; mean MMSE-J: 23.8 ± 3.8, with 64.7% clustered in the MCI-to-mild-dementia range) were analyzed. Subjective STAI scores showed a non-significant decreasing trend with a moderate effect size (*p* = 0.088, r = 0.414). Conversely, the FAA index significantly increased post-intervention (*p* = 0.038, Cohen’s d = 0.55). No significant correlation was found between changes in STAI and FAA (r = −0.15, *p* = 0.562).

**Conclusion:**

A single session of SRT was associated with an increase in FAA; however, the uncontrolled design precludes causal attribution to the intervention. The dissociation between subjective reports and neurophysiological changes suggests that FAA may complement self-reported measures by capturing aspects of acute emotional change that may be difficult to verbalize in people with cognitive impairment. The non-significant but moderate-sized reduction in STAI scores warrants confirmation in larger controlled studies.

## 1 Introduction

With the global aging of the population, maintaining the mental health of older adults has become an important social challenge. The number of people living with dementia is projected to increase substantially worldwide, from 57.4 million in 2019 to 152.8 million by 2050 (GBD 2019 Dementia Forecasting Collaborators, 2022). Mild cognitive impairment (MCI) has been documented to exhibit a remarkably high worldwide prevalence among community-dwelling older adults (Bai et al., 2022). In addition to cognitive decline, psychological symptoms such as anxiety are also clinically relevant in later life. Anxiety disorders are prevalent among community-dwelling older adults (Gum et al., 2009). Among people with dementia, anxiety may occur as one of the neuropsychiatric symptoms commonly referred to as behavioral and psychological symptoms of dementia (BPSD) (Lyketsos et al., 2002). However, the present study did not assess BPSD as a syndrome; rather, it focused specifically on immediate changes in state anxiety among older adults with a range of cognitive function levels. Anxiety has been associated with an increased risk of cognitive impairment and dementia in community-dwelling older adults (Gulpers et al., 2016). Therefore, developing feasible non-pharmacological approaches to anxiety for older adults with diverse cognitive function levels is an important clinical and research priority.

Because pharmacological treatments for anxiety and other neuropsychiatric symptoms may carry risks of side effects in older adults, interest in non-pharmacological therapies has grown in recent years. Reminiscence therapy (RT), a representative method, is a psychosocial intervention that contributes to enhancing self-esteem and reducing depression and anxiety by intentionally recalling past events and experiences (Butler, 1963). A Cochrane systematic review reported that RT may provide small benefits in quality of life, cognition, communication, and mood for people with dementia; however, effects were inconsistent and varied across settings and delivery formats (Woods et al., 2018). Although other meta-analyses have shown that RT is particularly effective in reducing depressive symptoms (Pinquart and Forstmeier, 2012; Saragih et al., 2022), findings regarding its effects on anxiety symptoms remain inconsistent (Pinquart and Forstmeier, 2012).

In recent years, technological advancements have been significantly changing the form of RT delivery. In particular, immersive virtual reality-based reminiscence therapy (VRRT) has been shown to be highly feasible for older adults (Benoit et al., 2015). Furthermore, recent randomized controlled evidence indicates that immersive VRRT can elicit significant user engagement, improve behavioral and psychological symptoms, and enhance emotional well-being in people with dementia compared to conventional methods (Pereira et al., 2025). The high sense of immersion provided by VR is thought to effectively capture participants’attention, thereby facilitating deeper emotional engagement and narrative recall during the reminiscence process (Pereira et al., 2025). Furthermore, multisensory stimulation (MSS) is known to elicit relaxation effects and positive emotional reactions by directly stimulating sensory organs without involving intellectual activity, making it applicable even to individuals with severe dementia (Sánchez et al., 2013; Calderone et al., 2025).

“Smart Reminiscence Therapy (SRT),” proposed in this study, is a new intervention model that integrates the concepts of VRRT and MSS. SRT synchronizes an immersive visual experience via VR with multiple sensory stimuli, such as sound (auditory) and scent (olfactory). This multisensory approach aims for a more physical and emotional “re-experiencing” of past events, rather than mere memory recall. It is based on the neuroscientific hypothesis that the integration of sensory information more strongly activates brain regions associated with emotion (e.g., the amygdala and insula cortex), leading to more powerful emotional arousal and anxiety-reduction effects than single sensory stimulation alone.

One of the greatest challenges in intervention studies targeting older adults is the reliability of outcome measurements. Subjective self-report scales like the State-Trait Anxiety Inventory (STAI) require high-level cognitive functions, such as understanding the questions. Thus, their reliability may be compromised depending on the cognitive level of the participants (Kinchin et al., 2026). To overcome this challenge, this study adopted frontal alpha asymmetry (FAA) calculated from electroencephalography (EEG) as an objective physiological measure. FAA indexes the difference in alpha wave power between the left and right frontal regions and is thought to reflect the neural substrates of emotional states and motivation (Davidson, 1998; Reznik and Allen, 2018). According to the approach-withdrawal model by Davidson and colleagues, the left frontal region is involved in positive emotion and approach motivation, while the right frontal region is involved in negative emotion and withdrawal motivation (Davidson, 1998). Because EEG alpha power is inversely correlated with cortical activity, as demonstrated by electrophysiological and functional neuroimaging studies (Laufs et al., 2003), a state of relatively higher right frontal activity (lower right frontal alpha power) is considered to reflect negative emotional states such as anxiety and depression (Thibodeau et al., 2006). Indeed, it has been reported that patients with anxiety disorders exhibit relatively higher right frontal activity at rest (Thibodeau et al., 2006), and interventions such as cognitive behavioral therapy (CBT) have been shown to shift this asymmetry toward a left-frontal-dominant pattern (Moscovitch et al., 2011).

This study was positioned as an exploratory pilot study to obtain preliminary information regarding immediate changes following SRT and the feasibility of electrophysiological measurement in older adults with diverse cognitive function levels. The objectives were (1) to obtain preliminary evidence on whether a single session of SRT brings about an immediate reduction effect on anxiety in older adults with a wide range of cognitive levels, including those from healthy to cognitively impaired, and (2) to evaluate whether FAA can serve as an objective and feasible measure for assessing intervention effects in this diverse older population.

We hypothesized the following in this study:

1. Following the SRT intervention, participants’self-reported state anxiety (STAI scores) will decrease.
2. Following the SRT intervention, FAA will significantly increase. This reflects a decrease in right frontal activity or an increase in left frontal activity, providing neurophysiological evidence of anxiety reduction.

## 2 Methods

### 2.1 Study design

This study adopted a single-session, single-arm pre-post exploratory design to perform a preliminary evaluation of the intervention effects and to examine the feasibility of the measurement methods.

### 2.2 Participants

The participants were 20 older adults utilizing a specific day service center in Osaka Prefecture.

Inclusion Criteria: Older adults aged 65 years or older were targeted. In this study, to reflect the diversity of users in actual adult day care (day service) settings and to verify the broad applicability of the intervention, the inclusion criteria encompassed older adults with a wide range of cognitive function levels, from the cognitively intact range (30 points) to moderate cognitive impairment (11 points).

Exclusion Criteria: Mini-Mental State Examination-Japanese (MMSE-J) score of 10 points or less. Severe paralysis or severe sensory impairment (visual, olfactory, or auditory).

Since this study is a pilot trial, a sample size design based on *a prior* power analysis for the primary outcomes was not performed. However, the sample size of this study (target enrollment N = 20, final analysis sample N = 17) satisfies the scale recommended for exploratory studies (12 to 35 individuals per arm) aimed at evaluating the feasibility of an intervention and estimating statistical parameters (such as standard deviation and effect size) for future main trials (Julious, 2005; Whitehead et al., 2016). Additionally, the 15% (3/20 participants) data attrition rate that occurred in this study (exclusion from analysis due to excessive EEG artifacts) provides preliminary information for estimating potential attrition when planning future controlled studies involving older adults with cognitive impairment.

### 2.3 Ethical considerations

This study protocol was approved by the Research Ethics Committee of the Graduate School of Rehabilitation Sciences, Osaka Metropolitan University (Approval Number: 2022-201). All procedures complied with the ethical principles of the Declaration of Helsinki (World Medical Association, 2013). Because participants with cognitive impairment represent a vulnerable population, special attention was paid to the informed consent process. Prior to the study sessions, the researcher gave each participant a presentation and explained verbally in plain language the purpose, procedures, benefits, and risks of the study, as well as the right to withdraw from participation at any time. Written informed consent was obtained directly from participants who were judged to have sufficient decision-making capacity. When decision-making capacity was questionable, informed consent was obtained from a legal representative as a proxy, in addition to obtaining the participant’s assent (Black et al., 2007).

### 2.4 Smart reminiscence therapy (SRT) intervention

An all-in-one SRT system that integrated and stimulated three senses (visual, auditory, and olfactory) was used for the intervention (Figure 1).

**FIGURE 1:**
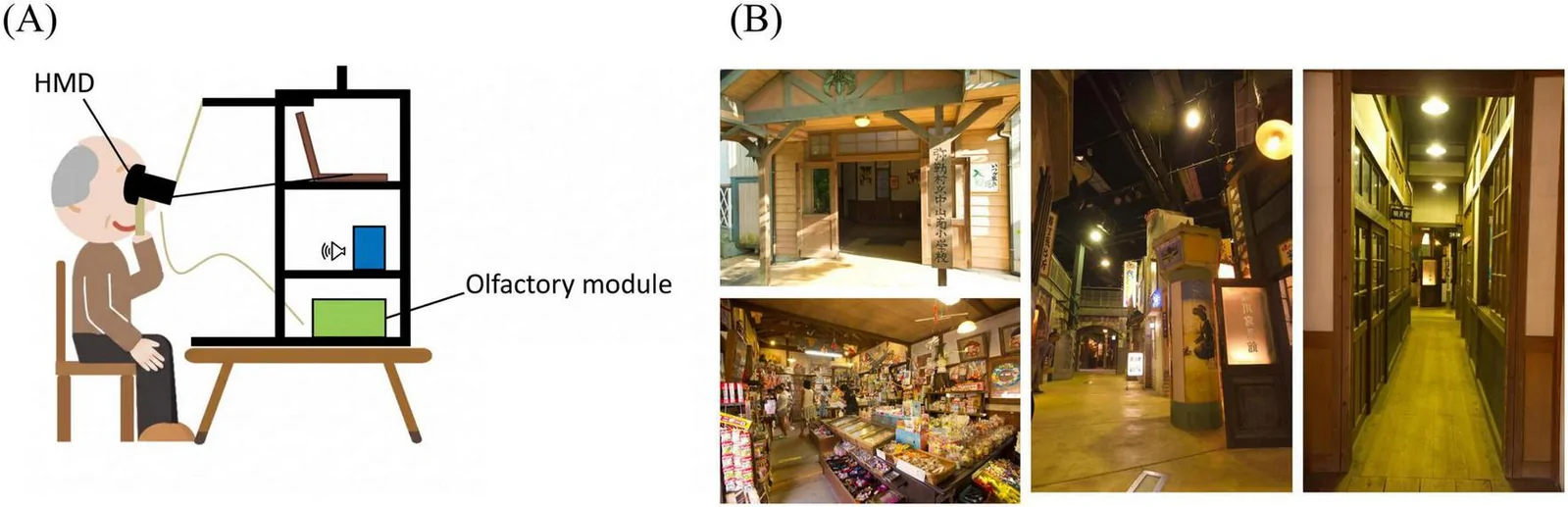
Overview and visual content of the SRT system. **(A)** Schematic illustration of the all-in-one SRT system designed to deliver synchronized multisensory stimuli, including immersive visual, auditory, and olfactory stimuli. **(B)** A representative screenshot of the 360-degree VR visual content depicting a replicated 1960s Japanese townscape utilized to facilitate spontaneous reminiscence. VR, virtual reality; SRT, smart reminiscence therapy. Photographs in panel **(B)** were provided by Miroku-no-Sato (©Miroku-no-Sato) and are reproduced with permission. These photographs are not covered by the article’s CC BY license.

#### 2.4.1 Visual stimulation

Participants wore a VR head-mounted display (HMD; EYE THEATER FOCUS, Thanko Co., Ltd., Tokyo, Japan). As VR content, high-definition 360-degree real-world video clips of “Miroku-no-sato,” a theme park replicating a 1960s Japanese townscape (including a wooden elementary school, a coffee shop, and a bar), were presented (Miroku-no-Sato, n.d.). Before the SRT session, a researcher orally explained the available virtual locations to each participant. Participants were asked to select the location they wished to view based on their preferences. When a participant did not express a specific preference, the location was selected collaboratively through conversation between the participant and the researcher. During the session, participants generally viewed a single selected location or environment. The researcher operated the viewpoint within the 360-degree video using a joystick, enabling movement through the selected virtual environment.

#### 2.4.2 Auditory stimulation

Environmental sounds synchronized with the VR video were provided through a speaker.

#### 2.4.3 Olfactory stimulation

Fragrances blended to match the scenes of the video were provided from a device (Aroma Shooter^®^, Aromajoin Inc.) built into the system (e.g., Japanese cypress scent corresponding to the wooden elementary school video, coffee scent corresponding to the coffee shop video, and whiskey scent corresponding to the bar video).

#### 2.4.4 Interactive approach by the researcher (facilitation of reminiscence)

During the 10-min SRT intervention session, at least one researcher with clinical experience was always seated to the side of the participant. While managing the participant’s safety, the researcher implemented verbal interventions (facilitation) to encourage spontaneous memory recall (reminiscence) induced by the VR video and multisensory stimulation. Specifically, for spontaneous narratives uttered by the participants (e.g., “Nostalgic, I used to go to a coffee shop like this”), dialogue based on the principles of active listening, empathy, and non-directive questioning (e.g., “What kind of memories do you have?” “Who did you go with?”) was conducted. Furthermore, even when spontaneous utterances were not observed, the researcher refrained from throwing forced questions to avoid interfering with the immersion or causing excessive cognitive load, maintaining a receptive involvement tailored to the participant’s pace.

The total duration of the SRT intervention was uniformly set to 10 min and 00 s per session. However, a protocol was established to immediately remove the HMD and discontinue the intervention if a participant verbally expressed discomfort or if abnormalities such as sudden body movements were observed (notably, there were no dropouts who discontinued the intervention mid-course in this study).

### 2.5 Experimental procedures and outcome measures

After arriving at the day service center, participants received a standardized explanation of the study procedures from a researcher before the assessments began. All assessments and intervention sessions were conducted in the same assessment environment. The procedures were performed in a fixed order for all participants. First, state anxiety was assessed using the STAI. Next, resting-state eye-closed EEG was recorded. Participants then received the 10-min SRT intervention. Immediately after the intervention, resting-state eye-closed EEG was recorded again, followed by a post-intervention assessment of state anxiety using the STAI. Finally, adverse events, including headache, nausea, and dizziness, were assessed using a 10-point numerical rating scale (NRS) and verbal confirmation.

#### 2.5.1 Cognitive function

Assessed within one month prior to the start of the experiment using the MMSE-J.

#### 2.5.2 Primary outcome (FAA)

Frontal brain activity was measured using a wearable EEG device “HARU” (PGV Inc.). Resting-state eye-closed EEG was measured for 2 min and 30 s both before and after the intervention to calculate FAA. During the recording, participants were explicitly instructed to remain seated as still as possible, close their eyes, and relax without focusing on any specific thoughts.

#### 2.5.3 Secondary outcome (STAI)

Subjective state anxiety was assessed before and after the intervention using the state anxiety scale of the STAI Form Y-1 (Hidano et al., 2000).

#### 2.5.4 Adverse events

Items including headache, nausea, and dizziness were assessed after the intervention using a 10-point NRS, and the presence or absence of adverse events was confirmed verbally.

### 2.6 EEG recording and analysis

Recording settings: Electrodes were placed at the frontal pole regions (Fpz, Fp1, Fp2) based on the International 10–20 system. Although most previous studies on FAA have reported findings in posterior frontal regions such as F3/F4 or F7/F8 (Allen et al., 2004; Reznik and Allen, 2018), recent meta-analytic reviews of resting-state FAA have increasingly incorporated studies utilizing frontopolar montages (Fp1/Fp2), reflecting an expanding application of these sites in screening for major depression (Luo et al., 2025), which characteristically shares overlapping neural dimensions with broader affective and anxiety processing (Allen et al., 2018). Furthermore, in clinical monitoring settings, preoperative resting alpha asymmetry calculated directly from Fp1 and Fp2 sensors has been demonstrated to be significantly associated with patients’ psychological distress and subsequent emotional recovery trajectories (Bae et al., 2023). Based on these precedents, this study adopted an easy-to-wear frontal pole wearable EEG device. This choice was intentionally made to minimize physical and psychological burden on oldest-old individuals with cognitive impairment, thereby optimizing clinical feasibility and practicality in actual adult day care (day service) environments. The reference electrode was placed on the left mastoid, the sampling rate was set to 250 Hz, and the bandpass filter was set to 4.0–50.0 Hz.

Pre-processing and artifact removal: BESA Research 7.1 (BESA GmbH) was used for EEG analysis. After segmenting continuous data into 2.05-s epochs, rigorous artifact removal was implemented. First, a trained researcher manually removed epochs containing body movements and eye movements by visual inspection. Next, using the automatic artifact scanning function of BESA Research, epochs containing artifacts were removed based on objective criteria (amplitude threshold: 120 microvolts). Due to these rigorous criteria, three participants with insufficient data quality were excluded from the analysis.

Fast Fourier Transform (FFT) was applied to the epochs after artifact removal to calculate the absolute power in the alpha band (8–14 Hz) at the Fp1 and Fp2 electrodes. Although traditional protocols generally used a narrower window of 8.0–13.0 Hz (Reznik and Allen, 2018), a broader range up to 14.0 Hz was applied here to capture the wider alpha spectrum. To minimize the effect of individual differences in overall EEG amplitude, the FAA values were calculated as a normalized asymmetry index using the absolute power in the alpha band at the Fp1 and Fp2 electrodes. Specifically, the following formula was applied: FAA = (alpha power at Fp2 – alpha power at Fp1)/(alpha power at Fp2 + alpha power at Fp1). Higher positive values indicate a relatively higher right frontal alpha power, reflecting relative left frontal cortical activity.

### 2.7 Statistical analysis

IBM SPSS Statistics (Version 28.0) was used for statistical analysis. The paired *t*-test was used to analyze the primary outcome (FAA values), and the Wilcoxon signed-rank test was used to evaluate the secondary outcome (STAI scores) before and after the intervention. The statistical significance level was set at alpha = 0.05 (two-tailed) for all analyses. To examine the relationship between the amount of change in FAA and the amount of change in STAI scores, Spearman’s rank correlation coefficient was calculated.

## 3 Results

### 3.1 Participant characteristics

The final analysis included 17 participants. Their demographic and clinical characteristics are shown in Table 1. Regarding the inclusion criteria (MMSE-J: 11–30 points), the actual distribution of MMSE-J scores among the 17 participants was clustered within the range of 20 to 27 points (MCI to mild dementia level), accounting for 64.7% (11 individuals) of the total. Participants falling into moderate dementia at 19 points or less (2 individuals) or the cognitively intact level at 28 points or higher (4 individuals) were a minority.

**TABLE 1:** Participant Baseline Characteristics (N = 17).

n = 17	Baseline
Age	85.9 ± 5.9
Gender	female: 9
MMSE	23.8 ± 3.8

Values are presented as mean ± SD for continuous variables, or as counts for categorical variables. MMSE, Mini-Mental State Examination; SD, standard deviation.

### 3.2 Subjective and physiological effects of the SRT intervention

#### 3.2.1 Physiological anxiety marker (FAA)

Following the intervention, the FAA value significantly increased (pre-intervention: 0.0077 ± 0.053, post-intervention: 0.0438 ± 0.061; *p* = 0.038, Cohen’s d = 0.55). This result indicates that relative left frontal activity was enhanced after the SRT intervention (Figure 2 and Table 2).

**FIGURE 2:**
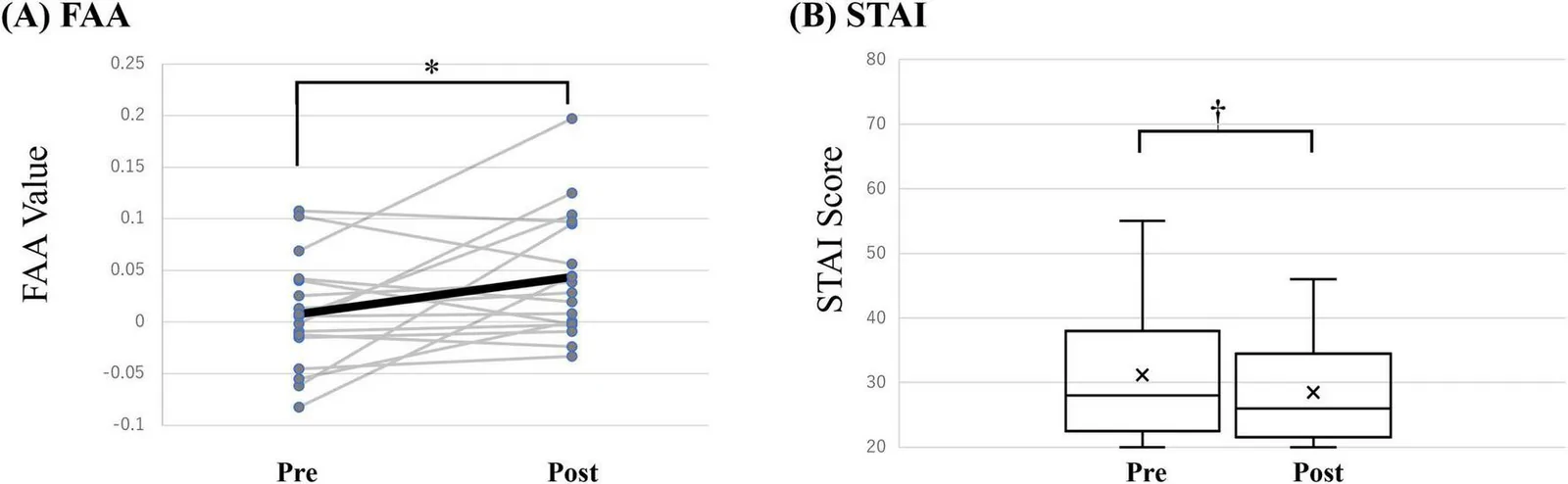
Changes in FAA and STAI following the SRT intervention. **(A)** Individual trajectory plot for FAA, where fine lines represent unique individual shifts between pre- and post-intervention states, and the bold line highlights the overall mean trajectory. The FAA index significantly increased post-intervention. **(B)** Box-and-whisker plot for STAI scores representing the median and interquartile range, demonstrating a marginal decrease under a baseline floor effect. FAA, frontal alpha asymmetry; STAI, State-Trait Anxiety Inventory; SRT, smart reminiscence therapy. ^†^*p* < 0.10, *p < 0.05.

**TABLE 2:** Statistical comparison of subjective anxiety scores (STAI) and frontal alpha asymmetry (FAA) before and after the SRT intervention.

Outcome measure	Pre	Post	*p* value
FAA	0.0077 ± 0.053	0.0438 ± 0.061	*p* = 0.038*
STAI	31.18 ± 10.36	28.47 ± 8.73	*p* = 0.088^†^

Values are presented as mean ± SD. FAA data were analyzed using the paired *t*-test, whereas STAI scores were analyzed using the Wilcoxon signed-rank test. SD, standard deviation; STAI, State-Trait Anxiety Inventory; FAA, frontal alpha asymmetry; SRT, smart reminiscence therapy.

† *p* < 0.10, *p < 0.05.

#### 3.2.2 Subjective anxiety (STAI)

The mean STAI state anxiety score was 31.18 ± 10.36 before the intervention and 28.47 ± 8.73 after the intervention. The corresponding median (Q1–Q3) values were 28 (23–35) and 26 (22–34), respectively. STAI scores decreased following the intervention; however, this change did not reach statistical significance (*p* = 0.088, r = 0.414) (Figure 2 and Table 2).

#### 3.2.3 Relationship between subjective and physiological measures

No significant correlation was observed between the amount of change in the STAI score (post-intervention – pre-intervention) and the amount of change in the FAA value (post-intervention – pre-intervention) due to the intervention (Spearman’s r = −0.15, *p* = 0.562).

#### 3.2.4 Adverse events

No participant discontinued the SRT session. Post-intervention NRS scores for nausea and headache were 0 for all participants. One participant reported dizziness with an NRS score of 2, whereas the remaining participants reported no dizziness. No other adverse events were observed by the researchers or verbally reported by the participants.

## 4 Discussion

This pilot study is the first attempt to explore the immediate effects of SRT, which integrates VR and multisensory stimulation, on anxiety in older adults with cognitive impairment using objective neurophysiological measures. The primary finding was that a significant change in FAA, suggesting a reduction in anxiety, was observed after a single session of SRT.

### 4.1 Interpretation of neurophysiological changes

The most important finding of this study is that the FAA value significantly increased after the SRT intervention. This means an enhancement of relative left frontal activity, which, according to the approach-withdrawal model of emotion, reflects a decrease in negative emotion such as anxiety and avoidance motivation (Davidson, 1998; Reznik and Allen, 2018). In the normalized asymmetry index used in this study, a post-intervention increase in the FAA value indicates a shift toward a state where right frontal alpha power is relatively dominant over left frontal alpha power. Because EEG alpha power is inversely correlated with cortical activity, this result suggests that the SRT intervention effectively induced a relative inhibition of the right frontal cortex, which is typically hyperactive during states of anxiety and avoidance motivation (Mennella et al., 2017). This finding suggests that SRT does not merely entertain participants but may potentially modulate the activity of neural circuits related to anxiety. However, this neurophysiological interpretation must be conditioned by technical caveats inherent to frontopolar EEG recordings, particularly regarding potential ocular artifact contamination.

### 4.2 Consideration of the dissociation between physiological and subjective measures

In this study, while a significant change was observed in the objective physiological measure (FAA), no significant change was seen in the subjective self-report measure (STAI), and there was no correlation between the amounts of change in both measures (Spearman’s r = −0.15). Although the negative sign of this correlation coefficient aligns with the theoretical expectation – where a treatment-induced decrease in STAI scores (negative change) paired with an increase in FAA values (positive change) mathematically yields an inverse relationship – the negligible magnitude of this coefficient requires precise interpretation. This dissociation highlights the inherent challenges of emotional assessment in an older adult population with a wide range of cognitive function levels.

First, neurophysiological measures such as FAA capture subtle changes in emotional responses without going through conscious evaluation or verbalization, and therefore may be more sensitive than self-reports (Reznik and Allen, 2018). On the other hand, self-report scales like the STAI require sophisticated cognitive processes, such as introspecting one’s emotional state after an intervention, retaining it in short-term memory, and further verbalizing it after correctly understanding the intent of the questions. Considering that many participants in this study exhibited mild dementia or MCI, it is highly likely that due to the difficulty of these cognitive steps, variance (noise) occurred in the subjective report data, making it impossible to capture acute emotional changes caused by the intervention through subjective measures. Furthermore, because the STAI is a scale standardized based on young and adult populations, structural limitations – such as the occurrence of floor effects – where it cannot sensitively isolate the unique psychological backgrounds (non-psychiatric factors) associated with the physical frailty of older adults or the adult day care environment, could also be considered a reason for the failure to detect significant differences. In the future, the adoption of anxiety scales specific to older adults should be considered.

Second, there are limits to psychometric statistical power and the possibility of attenuation due to measurement timing. Since this study is a pilot trial, it cannot be denied that the statistical power may have been insufficient to detect statistically significant changes in STAI scores, which showed large individual differences (standard deviations). Although the decrease in STAI scores did not reach conventional statistical significance (*p* = 0.088), the corresponding effect size was r = 0.414, which may be regarded as being in the moderate range according to commonly used benchmarks for correlation-based effect sizes. This finding should be interpreted cautiously, particularly given the small sample size and exploratory nature of this pilot study. Nevertheless, the observed effect size may indicate a possible reduction in subjective state anxiety following SRT. Thus, although the direction of the observed change was consistent with our hypothesis that SRT would reduce self-reported state anxiety, the present findings do not provide statistically conclusive support for this hypothesis. The lack of statistical significance may reflect limited statistical power, substantial individual variability in STAI scores, or both. Further studies with larger samples and an appropriate control condition are needed to determine whether this apparent change represents a reproducible subjective anxiolytic effect. In addition, due to the procedures of this study, a 2-min and 30-s EEG measurement was interposed between the end of the SRT intervention and the response to the STAI, and it is also conceivable that the immediate reduction effect on subjective state anxiety had partially attenuated by the time of the questionnaire response, contributing to the lack of correlation.

These considerations show the limitations of relying solely on self-reports in clinical studies targeting dementia or MCI. Objective physiological biomarkers like FAA can serve as measures that complement subjective assessments, or may be more reliable indicators under certain circumstances.

### 4.3 Clinical implications

The present findings suggest that multisensory VR reminiscence was feasible for delivery in this setting and was generally well tolerated in this small sample. Its safety, effectiveness, and clinical utility require confirmation in larger controlled studies.

### 4.4 Limitations of this study

Although this pilot study provided important findings, several limitations must be considered when interpreting the results.

First, the absence of a control group is the greatest limitation. Because this study used a single-arm pre-post comparison design, it cannot distinguish whether the observed changes in FAA were due to the SRT intervention itself or simply due to the passage of time or attention from the researcher. Therefore, causal inferences regarding the effects of SRT cannot be made from the present findings.

Second, the small sample size (*n* = 17) limits the generalizability of the results. Although the MMSE-J inclusion range was broad (11–30) to reflect the diversity of users in adult day care settings, the analyzed participants were clustered primarily in the MCI-to-mild-dementia range. Therefore, the present study cannot rigorously evaluate the effects of SRT in cognitively intact older adults or in people with moderate or severe dementia, nor can it determine whether potential effects differ according to the severity of cognitive impairment.

Third, technical limitations of EEG measurement should be considered. The wearable EEG device used in this study recorded only from the frontal poles (Fp1, Fp2), and may not directly reflect activity in the F3/F4 or F7/F8 regions commonly used in traditional FAA studies. Because frontal pole sites are particularly susceptible to artifacts associated with eye movements and blinking, this study implemented rigorous data pre-processing combining visual checks by a trained researcher and automatic artifact scanning with an amplitude threshold of 120 microvolts. However, because spatial filtering techniques such as Independent Component Analysis (ICA) were not applied, residual ocular artifact contamination in the alpha band cannot be completely excluded. Thus, the significant increase in FAA may have been influenced by differences in ocular artifacts between the pre- and post-intervention states. For example, greater eyes-closed stillness or fewer micro-blinks after the intervention could have contributed to changes in measured asymmetry without necessarily reflecting a change in underlying cortical activity. In addition, the reliance on a single left mastoid reference introduces a critical structural limitation. Because the reference electrode was placed exclusively on the left side, the shorter physical distance to Fp1 relative to Fp2 may have caused asymmetric signal cancellation, and a relative overestimation of right-frontal alpha power. Consequently, we cannot rule out the possibility that the observed increase in FAA was partially driven by this montage-specific physical artifact rather than genuine neural modulation. Furthermore, the fixed alpha band of 8.0–14.0 Hz may not have adequately captured individual differences in peak alpha frequency. Aging, particularly in people with MCI and dementia, is associated with a slowing of peak alpha frequency (Babiloni et al., 2017; Park et al., 2024). Therefore, the fixed alpha window may have introduced measurement error by failing to capture individually shifted alpha activity or by incorporating low-frequency beta activity.

Fourth, potentially relevant confounding factors were uncontrolled. The possibility that medications routinely taken by participants (such as psychotropic drugs) affected EEG or mood cannot be excluded.

Fifth, intervention heterogeneity and experimenter effects may have influenced the findings. To facilitate reminiscence, participants viewed VR content depicting nostalgic locations selected according to their preferences. Although this person-centered approach is consistent with the clinical principles of reminiscence therapy, visual characteristics of the selected content, such as luminance and spatial frequency, and differences in the chemical properties of the fragrances may have influenced EEG activity. Furthermore, during the 10-min session, at least one researcher with clinical experience was involved as a facilitator conducting dialogue at the side. Because multiple experimenters implemented the intervention, the possibility that differences in the quality of facilitation or rapport building (experimenter effects) influenced the participants’ psychological and physiological responses cannot be excluded.

Sixth, the SRT used in this study is a “multisensory combined intervention” that integrates visual and auditory stimuli using VR video, olfactory stimulation via aroma, and further interactive facilitation by a researcher. Therefore, the present study design cannot identify or isolate the individual effects of these components, or determine which component or combination of components contributed to the observed changes in anxiety-related measures or FAA.

Seventh, a significant conceptual limitation lies in the extrapolation of foundational FAA models to the oldest-old population. To date, the vast majority of electrophysiological literature regarding resting-state FAA and emotional regulation has been established using healthy young adults or younger clinical cohorts without neurodegenerative pathology. Literature exploring FAA specifically within the context of oldest-old individuals or those with cognitive decline remains critically scarce. Given that the aging brain undergoes distinct neurophysiological shifts – such as the slowing of baseline alpha rhythms and altered cortical connectivity – applying young-adult frameworks to a cohort with a mean age of 85.9 years demands highly cautious interpretation.

Eighth, the psychometric stability and validity of FAA as a metric of acute emotional shifts remain highly debated in the electrophysiological literature. Methodological reviews and large-scale meta-analyses have highlighted that FAA scores are highly susceptible to temporal variations and can be heavily confounded by biological factors such as age and gender (Towers and Allen, 2009; van der Vinne et al., 2017). Therefore, the significant pre-to-post change observed in our small sample might not reflect a true neurophysiological modulation uniquely driven by the SRT intervention. The present findings should consequently be regarded as preliminary and hypothesis-generating.

### 4.5 Future directions

Future studies should use controlled designs to clarify whether the observed changes are attributable to SRT rather than to the passage of time, repeated assessment, VR exposure, multisensory stimulation, or researcher attention. Appropriate comparison conditions may include VR viewing without synchronized sensory stimulation, 2D video viewing, or conditions that systematically vary individual intervention components.

Studies with larger samples and prespecified stratification by cognitive function level are needed to examine whether the feasibility and potential effects of SRT differ among cognitively intact older adults, people with MCI, and people with mild-to-moderate dementia. Medication status should be systematically recorded and considered in the analysis. Follow-up assessments are also needed to examine whether any psychological or neurophysiological changes persist beyond the immediate post-intervention period.

From a neurophysiological perspective, future studies should use multi-channel EEG systems with EOG recording to improve the identification and correction of ocular artifacts. Standardized and reproducible electrode placement procedures, recording from more posterior frontal regions, using more robust reference procedures, and adopting analysis windows tailored to individual alpha frequency should also be considered to improve the stability and interpretability of FAA measurements in older adults with cognitive impairment.

Finally, future studies should further standardize the intervention protocol while preserving the person-centered nature of reminiscence therapy. Standardized facilitator training, more structured guidance for dialogue, and systematic documentation of selected VR content and olfactory stimuli may reduce intervention-related variability. Component-based study designs will also be important to clarify the individual and combined contributions of VR, auditory stimulation, olfactory stimulation, and researcher facilitation.

## 5 Conclusion

This pilot study provides preliminary, hypothesis-generating evidence that SRT, which integrates VR and multisensory stimulation, may modulate neurophysiological activity associated with anxiety in older adults with a wide range of cognitive function levels. The significant increase in FAA observed after the SRT intervention may be consistent with reduced anxiety, although methodological limitations preclude definitive causal interpretation. These results suggest that FAA warrants further investigation as a potential objective biomarker that may complement self-reported measures for capturing changes in emotional states induced by interventions in populations with cognitive decline. These findings provide preliminary, hypothesis-generating information to support further refinement and controlled evaluation of the SRT protocol.

## Data Availability

The datasets generated and analyzed in this study are not publicly available due to ethical and privacy restrictions. Requests to access the datasets should be directed to the corresponding author, RI (ishii@psy.med.osaka-u.ac.jp). Any data sharing would be subject to applicable ethical and privacy requirements.
